# Plant-based diet adherence is associated with metabolic health status in adults living with and without obesity

**DOI:** 10.1007/s00394-024-03399-7

**Published:** 2024-05-16

**Authors:** Mags T. Carey, Seán R. Millar, Patrick S. Elliott, Pilar Navarro, Janas M. Harrington, Ivan J. Perry, Catherine M. Phillips

**Affiliations:** 1 School of Public Health, Physiotherapy and Sports Science, University College Dublin, Dublin 4, Ireland; 2 HRB Centre for Health and Diet Research, School of Public Health, University College Cork, Cork, Ireland; 3 Institute of Food and Health, School of Agriculture and Food Science, University College Dublin, Dublin 4, Ireland

**Keywords:** Plant-based diet, Metabolic health, Obesity, Dietary patterns, Metabolic phenotype

## Abstract

**Purpose:**

Metabolic health phenotypes exist across the body mass index spectrum. Diet may be an important modifiable risk factor, yet limited research exists on dietary patterns in this context. We investigated associations between dietary patterns, reflecting dietary quality, healthfulness and inflammatory potential, and metabolic health phenotypes in adults living with and without obesity.

**Methods:**

This cross-sectional study included 2,040 middle- to older-aged men and women randomly selected from a large primary care centre. The Dietary Approaches to Stop Hypertension score, Healthy Eating Index, Dietary Inflammatory Index, overall, healthful and unhealthful plant-based dietary indices and Nutri-Score were derived from validated food frequency questionnaires. Descriptive and logistic regression analyses were used to examine diet score relationships with metabolic health phenotypes (Metabolically Healthy/Unhealthy Obese (MHO/MUO) and Non-Obese (MHNO/MUNO)), defined using three separate metabolic health definitions, each capturing different aspects of metabolic health.

**Results:**

In fully adjusted models, higher unhealthful plant-based dietary scores were associated with a lower likelihood of MHO (OR = 0.96, 95% CI: 0.93–1.00, *p* = 0.038) and MHNO (OR = 0.97, 95% CI: 0.95–0.99, *p* = 0.006). Higher Nutri-Score values were associated with an increased likelihood of MHNO (OR = 1.06, 95% CI: 1.01–1.13, *p* = 0.033).

**Conclusion:**

These findings provide evidence that more unhealthful plant-based diets may be linked with unfavourable metabolic health status, irrespective of BMI.

**Supplementary Information:**

The online version contains supplementary material available at 10.1007/s00394-024-03399-7.

## Introduction

The prevalence of obesity has tripled worldwide over the last 40 years, representing a major public health issue, and it is estimated that more than one billion people will be living with obesity by 2030 [1]. Obesity is a complex and multifactorial disease, with lifestyle, genetic and environmental factors influencing its pathogenesis [2]. Obesity is also a primary risk factor for developing metabolic abnormalities including insulin resistance, dyslipidaemia and hypertension, and these metabolic abnormalities are associated with an increased risk of developing co-morbidities such as type 2 diabetes mellitus (T2DM), cardiovascular disease (CVD) and cancer [3]. Despite the increased risk of these co-morbidities, not all individuals living with obesity develop metabolic complications [4]. It has been estimated that these ‘metabolically healthy obese’ (MHO) individuals have half the risk of developing T2DM and CVD compared to those with a higher risk of metabolic abnormalities, i.e., the ‘metabolically unhealthy obese’ (MUO) [5]. Metabolic abnormalities may also exist in people living without obesity; the so-called metabolically unhealthy non-obese (MUNO). Therefore, improving our understanding of factors which promote or preserve metabolic health (MH) across the body mass index (BMI) spectrum is of paramount importance [6].

Modifiable lifestyle factors, such as diet, may have an impact on the pathogenesis of MH phenotypes. One study highlighted that higher adherence to healthier dietary patterns such as the Mediterranean diet (MD), abundant in antioxidant- and fibre-rich fruits and vegetables, may have a protective effect against adverse metabolic change and prevent the transition from MHO to an unhealthy metabolic state [7]. Conversely, unhealthy Western dietary patterns, characterised by the consumption of refined and ultra-processed foods such as fast food and sugar-sweetened beverages, have been associated with adverse MH changes and an increased risk of MUO [8]. However, current research in the context of MH phenotypes is limited in terms of dietary patterns and phenotypes investigated, with a majority of studies focusing on the MD [9, 10]. In addition, few studies have compared dietary pattern associations between obese and non-obese phenotypes which could have implications in terms of stratified or personalised preventive approaches, regardless of weight status. Furthermore, with the growing importance of shifting towards more plant-based diets (PBDs) for planetary health [11], expanding this field of research to incorporate PBDs is warranted.

This study addresses these gaps in the knowledge base. Using a random sample of 2,040 middle- to older-aged men and women, we conducted a comprehensive investigation of the relationships between eight dietary indices and obese and non-obese MH phenotypes (MHO/MUO and MHNO/MUNO), defined according to three definitions (MeigsA, MeigsB and Wildman) [12], to test the hypothesis that specific dietary patterns are related to MH status.

## Methods

### Study design and subject recruitment

The Cork and Kerry Diabetes and Heart Disease Study (Phase II) was a single centre, cross-sectional study conducted between 2010 and 2011 [13]. A population-representative random sample was recruited from a large primary care centre in Mitchelstown, County Cork, Ireland (Mitchelstown cohort). The Living Health Clinic includes eight general practitioners and serves a catchment area of approximately 20,000 with a mix of urban and rural residents. Mitchelstown cohort participants were randomly selected from all registered attending patients in the 46–73-year age group. In total, 3,807 potential participants were selected from the practice list. Following exclusion of duplicates, deaths and ineligibles, 3,043 were invited to participate in the study and of these, 2,047 Caucasian individuals (49% male) completed the questionnaire and physical examination elements of the baseline assessment (response of 67%). For this study, seven participants were excluded due to missing BMI data. Ethics committee approval conforming to the Declaration of Helsinki was obtained from the Clinical Research Ethics Committee of University College Cork. All participants provided written informed consent to use their data for research purposes.

### Dietary assessment

A modified version of the self-completed European Prospective Investigation into Cancer and Nutrition (EPIC) food frequency questionnaire (FFQ) was used for dietary assessment. Adapted to reflect the Irish population, the 150-item semi-quantitative FFQ used in the current study was originally validated for use in the Irish population using food diaries and a protein biomarker in a volunteer sample [14], and was incorporated into the SLÁN Irish National Surveys of Lifestyle Attitudes and Nutrition 1998, 2002 and 2007 [14–16]. The average medium serving of each food item consumed by participants over the last 12 months was converted into quantities using standard serving sizes of food items (grams per day) or beverages (millilitres per day). Daily intake of energy and nutrients was computed from FFQ data using a tailored computer programme (FFQ Software Version 1.0; developed by the National Nutrition Surveillance Centre, School of Public Health, Physiotherapy and Sports Science, University College Dublin, Belfield, Dublin 4, Ireland), which linked frequency selections with the food equivalents in McCance and Widdowson Food Table [17].

Based on the FFQ, eight dietary indices were calculated (see online Supplementary File for details on each scoring system), including: the DASH score [18, 19]; the Healthy Eating Index-2015 (HEI-2015) [20]; the Dietary Inflammatory Index (DII) [21, 22]; the Energy-Adjusted Dietary Inflammatory Index (E-DII); an overall plant-based diet index (PDI); a healthful plant-based diet index (hPDI); an unhealthful plant-based diet index (uPDI) [23, 24]; and the Nutri-Score [25]. For the DASH score and HEI-2015, lower scores represent a poorer quality diet whereas higher scores represent a higher quality diet. For both the DII and E-DII, higher scores are more pro-inflammatory and lower scores are more anti-inflammatory. For the PDI, higher scores represent a more PBD. For the hPDI, higher scores represent a more healthful PBD. For the uPDI, higher scores represent a more unhealthful PBD. For the Nutri-Score, higher scores reflect lower nutritional quality in the total foods consumed.

### Data collection, clinical measurements and classification of variables

A general health and lifestyle questionnaire assessed demographic variables and lifestyle behaviours. Information on age, sex, smoking status and alcohol use was provided by participants. Physical activity levels were measured using the validated International Physical Activity Questionnaire (IPAQ) [26]. Anthropometric measurements were recorded with calibrated instruments according to a standardised protocol. Body weight was measured in kilograms without shoes to the nearest 100 g using a Tanita WB100MA weighing scale (Tanita Corporation, IL, USA). Height was measured in centimetres to one decimal place using a Seca Leicester height gauge (Seca, Birmingham, UK). Waist circumference (defined as midway between lowest rib and iliac crest) was measured in centimetres to one decimal place using a Seca 200 measuring tape (Seca, Birmingham, UK). The average of two measurements was used for analyses. BMI was calculated, with values < and ≥ 30 kg/m^2^ indicating the absence or presence of obesity, respectively.

Smoking status was defined as follows: (i) never smoked, i.e., having never smoked at least 100 cigarettes (5 packs) in their entire life; (ii) former smoker, i.e., having smoked 100 cigarettes in their entire life and do not smoke at present; and (iii) current smoker, i.e., smoking at present. These definitions were the same as those used in the SLÁN Irish National Survey of Lifestyle Attitudes and Nutrition 2007 [27]. A variable was then created to reflect these definitions: ‘never’, ‘former smoker’ and ‘current smoker’, respectively. Alcohol consumption was measured in units of alcohol consumed on a weekly basis and was categorised into the following levels: (i) non-drinker, i.e., < 1 drink per week; (ii) moderate drinker, i.e., between 1 and 14 drinks per week; and (iii) heavy drinker, i.e., > 14 drinks per week. The definition of ‘moderate drinker’ was taken from the EPIC-Norfolk study [28]. Physical activity was categorised as low, moderate and high levels of activity using the IPAQ.

MH status was defined using three previously described MH phenotype definitions (Supplementary Table S1) [12]: MeigsA (metabolic syndrome phenotype); MeigsB (insulin resistance phenotype); and Wildman (metabolic syndrome plus inflammatory phenotype). The four phenotypes investigated were MHO, MUO, MHNO and MUNO [12].

### Statistical analysis

Descriptive characteristics and dietary parameters were examined for the population according to MH status (MHO, MUO, MHNO and MUNO) defined by the MeigsA, MeigsB and Wildman definitions (Supplementary Table S1). Continuous variables are expressed as means (± SD) and categorical variables are shown as counts and percentages. Differences between phenotypes within the same BMI category (MHO vs. MUO and MHNO vs. MUNO) were evaluated using independent samples t-tests or a one-way analysis of variance for continuous variables and Pearson’s chi-square tests for categorical variables. Logistic regression analyses assessed associations between dietary scores and the likelihood of MH phenotypes. Three models were run: an unadjusted model (Model 1), an age- and sex-adjusted model (Model 2) and a model additionally adjusted for energy intake, smoking status, alcohol use and physical activity (Model 3). Multivariable models examining the E-DII and Nutri-Score were not adjusted for energy intake as this is considered in the calculation of both scores. Sensitivity analyses were performed excluding participants with implausible energy intakes using sex-specific cut-offs of < 500 and > 3,500 kcal/d for females (*n* = 90), and < 800 and > 4,000 kcal/d for males (*n* = 68) [29]. For all analyses, a p-value (two-tailed) of less than 0.05 was considered to indicate statistical significance. To correct for the multiple testing performed in logistic regression analyses and reduce the risk of Type I errors, we applied the Benjamini and Hochberg correction for multiple testing with a false discovery rate of 0.05 [30]. All analyses were conducted using SPSS version 27.0 (IBM Corporation, Armonk, NY, USA) for Windows. Figures were created in RStudio version 4.3.0 (Posit Software) using the ggplot2 package from the tidyverse suite of packages [31].

## Results

### Descriptive characteristics

Characteristics of study participants (*n* = 2,040) according to MH definitions are presented in Table 1. The prevalence of MH phenotypes varied considerably between definitions. Among adults with obesity, the prevalence of MHO ranged from 24% (Wildman) to 37% (MeigsB) and MUO ranged from 63% (MeigsB) to 76% (Wildman). Among adults without obesity, the prevalence of MHNO ranged from 58% (Wildman) to 78% (MeigsB) and MUNO ranged from 22% (MeigsB) to 42% (Wildman). The prevalence of metabolically unhealthy phenotypes was higher in males than in females for MeigsB and Wildman definitions, with MUO ranging from 58% (Wildman) to 61% (MeigsB) and MUNO ranging from 54% (Wildman) to 61% (MeigsB). Participants presenting with metabolically healthy phenotypes had a lower BMI and waist circumference than their metabolically unhealthy counterparts, and this was statistically significant across all phenotype definitions.

**Table 1:** Characteristics of the study population according to metabolic health phenotype

Variable	MeigsA						MeigsB						Wildman					
n (%) 2,040	202 (30.3)	466 (69.7)		928 (67.6)	444 (32.4)		234 (36.6)	405 (63.4)		1036 (78.2)	289 (21.8)		158 (23.7)	510 (76.3)		800 (58.3)	527 (41.7)	
	**MHO**	**MUO**	**p-value**	**MHNO**	**MUNO**	**p-value**	**MHO**	**MUO**	**p-value**	**MHNO**	**MUNO**	**p-value**	**MHO**	**MUO**	**p-value**	**MHNO**	**MUNO**	**p-value**
Age (years)	59.3 (5.7)	60.4 (5.3)	**0.01**	58.7 (5.4)	61.4 (5.1)	**< 0.001**	60.1 (5.6)	60.0 (5.3)	0.735	59.4 (5.5)	60.5 (5.6)	**0.003**	60.0 (6.0)	60.1 (5.3)	0.795	58.5 (5.3)	61.1 (5.6)	**< 0.001**
Sex (%)																		
Male	50.5	57.1	0.116	45.5	48.2	0.325	43.2	61.2	**< 0.001**	42.3	60.6	**< 0.001**	46.8	57.6	**0.017**	40.9	53.8	**< 0.001**
Female	49.5	42.9		54.6	51.8		56.8	38.8		57.7	39.4		53.3	42.4		59.1	46.2	
Education (%)																		
Primary	29.1	34.7	0.281	23.4	30.7	**0.001**	28.2	35.0	0.071	24.1	29.2	0.208	28.9	34.3	0.46	21.1	32.3	**< 0.001**
Secondary	52.9	46.2		49.2	50.1		55.1	45.3		50.6	48.5		51.7	47.2		50.4	48.2	
Tertiary	18.0	19.0		27.4	19.2		16.7	19.7		25.3	22.3		19.5	18.5		28.5	19.5	
BMI (kg/m^2^)	33.0 (2.9)	34.2 (3.9)	**< 0.001**	25.6 (2.6)	27.0 (2.3)	**< 0.001**	32.8 (3.0)	34.3 (3.7)	**< 0.001**	25.6 (2.6)	27.7 (1.9)	**< 0.001**	32.7 (3.0)	34.2 (3.8)	**< 0.001**	25.6 (2.6)	26.8 (2.5)	**< 0.001**
WC (cm)	105.9 (10.5)	110.6 (10.5)	**< 0.001**	89.3 (9.5)	94.9 (9.5)	**< 0.001**	105.0 (9.1)	111.3 (10.7)	**< 0.001**	89.5 (9.7)	96.6 (8.3)	**< 0.001**	105.4 (10.3)	110.4 (10.6)	**< 0.001**	89.1 (9.6)	94.0 (9.5)	**< 0.001**
Kilocalories	1969.5 (728.5)	2096.0 (827.9)	0.063	2015.5 (841.3)	2035.8 (783.6)	0.673	2059.6 (829.8)	2060.9 (794.3)	0.985	2043.0 (838.5)	1974.6 (736.5)	0.219	1984.3 (738.1)	2080.2 (818.3)	0.193	2030.4 (851.4)	2010.7 (782.0)	0.666
Smoking (%)																		
Never	7.1	11.6	0.231	53.7	49.8	0.384	50.7	48.6	0.669	53.1	51.6	0.21	47.4	49.6	**0.03**	54.3	49.9	0.263
Former	41.8	40.1		29.7	32.7		40.4	40.2		29.8	34.5		47.1	38.6		29.1	32.8	
Current	51.0	48.3		16.6	17.5		9.0	11.2		17.1	13.9		5.2	11.8		16.6	17.3	
Physical Activity (%)																		
Low	51.9	55.4	0.625	44.7	47.8	0.587	47.7	59.1	**0.024**	45.0	48.5	0.165	51.0	55.3	0.62	43.3	49.2	**0.012**
Moderate	28.6	27.9		30.6	29.3		31.4	25.6		30.0	32.1		30.9	27.3		33.4	25.7	
High	19.6	16.7		24.6	22.9		20.9	15.3		25.0	19.4		18.1	17.4		23.3	25.1	
Alcohol (%)																		
Non-drinker	20.9	24.2	0.464	19.2	17.6	0.557	24.1	23.0	0.799	18.3	19.7	0.661	22.8	23.3	0.909	18.8	18.6	0.954
Moderate and Heavy Drinker	79.1	75.8		80.8	82.4		75.9	77.0		81.7	80.3		77.2	76.7		81.2	81.4	

Abbreviations: BMI: Body Mass Index, MHNO: Metabolically Healthy Non-Obese; MHO: Metabolically Healthy Obese, MUNO: Metabolically Unhealthy Non-Obese, MUO: Metabolically Unhealthy Obese, WC: Waist Circumference

Continuous variables are presented as means ± SD.

Statistical analyses were conducted using Student’s t-test for continuous variables and Pearson’s chi-square test for categorical variables

Significant p-values are highlighted in **bold**

### Dietary score comparisons between phenotypes

Dietary scores for the four phenotypes, according to the three MH definitions, are presented in Table 2. Overall, comparing dietary scores across phenotypes, there were significant differences for the DASH score (MeigsB: *p* = 0.001 and Wildman: *p* = 0.011), DII (MeigsB: *p* = 0.013 and Wildman: *p* < 0.001), E-DII (MeigsB: *p* = 0.043), PDI (MeigsA, MeigsB and Wildman: all *p* < 0.001), hPDI (MeigsA, MeigsB and Wildman: all *p* ≤ 0.001) and uPDI (MeigsB and Wildman: both *p* < 0.001). Comparison of metabolically healthy vs. unhealthy phenotypes revealed some definition-specific differences. With regards to those living with obesity, individuals with MHO phenotypes had higher DASH scores (MeigsB: *p* = 0.012) and lower DII scores (MeigsB: *p* = 0.044, Wildman: *p* = 0.009), indicating higher dietary quality and a more anti-inflammatory diet, respectively, than their metabolically unhealthy counterparts. Among those without obesity, individuals with MHNO phenotypes also had higher DASH scores (MeigsB: *p* = 0.006, Wildman: *p* = 0.017) and lower DII scores (MeigsB: *p* = 0.013, Wildman, *p* = 0.002) than individuals with MUNO phenotypes. Regarding PBD scores, individuals with MHO phenotypes had higher overall PDI (MeigsA: *p* = 0.046) and hPDI scores (MeigsB: *p* = 0.017), reflecting a more PBD and healthful PBD, respectively, and lower uPDI scores (MeigsB: *p* = 0.014 and Wildman: *p* = 0.015), reflecting a less unhealthful PBD, than those with MUO phenotypes. Individuals with MHNO phenotypes had higher hPDI (MeigsB: *p* = 0.002) and consistently lower uPDI scores (for all three definitions) compared to their metabolically unhealthy counterparts (MeigsA: *p* = 0.034, MeigsB and Wildman: both *p* < 0.001). In addition, those with the MHNO phenotype had higher Nutri-Score values (MeigsA: *p* = 0.017) than their metabolically unhealthy counterparts, reflecting a diet of lower nutritional quality.

**Table 2:** Dietary indices according to metabolic health phenotypes among participants with and without obesity

Variable	Category	MeigsA	p-value	p-value*	MeigsB	p-value	p-value*	Wildman	p-value	p-value*
**DASH score**	MHO	26.8 (4.9)	0.143	0.089	27.1 (5.3)	**0.012**	**0.001**	26.8 (4.9)	0.226	**0.011**
	MUO	26.2 (5.3)			26.0 (5.1)			26.2 (5.2)		
	MHNO	27.0 (5.5)	0.41		27.1 (5.5)	**0.006**		27.2 (5.5)	**0.017**	
	MUNO	26.7 (5.4)			26.0 (5.3)			26.5 (5.4)		
**HEI-2015**	MHO	39.6 (6.8)	0.359	0.452	39.3 (6.8)	0.771	0.336	39.3 (6.8)	0.882	0.565
	MUO	39.1 (6.9)			39.1 (6.9)			39.2 (6.9)		
	MHNO	39.6 (7.2)	0.583		39.7 (7.1)	0.185		39.8 (7.3)	0.485	
	MUNO	39.7 (6.8)			39.1 (7.0)			39.5 (6.9)		
**DII**	MHO	-0.4 (1.6)	0.053	0.063	-0.4 (1.5)	**0.044**	**0.013**	-0.5 (1.4)	**0.009**	**< 0.001**
	MUO	-0.1 (1.6)			-0.1 (1.6)			-0.1 (1.6)		
	MHNO	-0.3 (1.6)	0.106		-0.3 (1.6)	**0.013**		-0.4 (1.6)	**0.002**	
	MUNO	-0.2 (1.6)			-0.1 (1.6)			-0.1 (1.6)		
**E-DII**	MHO	-0.7 (1.5)	0.876	0.281	-0.8 (1.4)	0.096	**0.043**	-0.7 (1.3)	0.975	0.493
	MUO	-0.7 (1.4)			-0.6 (1.5)			-0.7 (1.5)		
	MHNO	-0.8 (1.4)	0.204		-0.8 (1.4)	0.052		-0.8 (1.4)	0.639	
	MUNO	-0.9 (1.5)			-0.6 (1.5)			-0.8 (1.5)		
**PDI**	MHO	51.0 (6.1)	**0.046**	**< 0.001**	50.5 (5.6)	0.592	**< 0.001**	51.1 (6.0)	0.068	**< 0.001**
	MUO	50.0 (6.0)			50.3 (6.2)			50.1 (6.1)		
	MHNO	51.6 (5.9)	0.16		51.6 (5.9)	0.168		51.7 (5.8)	0.059	
	MUNO	51.2 (5.9)			51.0 (5.8)			51.1 (6.0)		
**hPDI**	MHO	51.8 (6.7)	0.767	**0.001**	52.8 (7.0)	**0.017**	**< 0.001**	52.1 (7.1)	0.804	**< 0.001**
	MUO	52.0 (6.9)			51.5 (6.8)			51.9 (6.8)		
	MHNO	53.2 (7.0)	0.539		53.5 (7.0)	**0.002**		53.5 (7.0)	0.268	
	MUNO	53.5 (7.5)			52.1 (7.7)			53.0 (7.3)		
**uPDI**	MHO	51.5 (7.4)	0.167	0.088	51.2 (6.8)	**0.014**	**< 0.001**	50.9 (7.2)	**0.015**	**< 0.001**
	MUO	52.4 (6.9)			52.7 (7.2)			52.5 (7.0)		
	MHNO	51.7 (7.3)	**0.034**		51.7 (7.1)	**< 0.001**		51.2 (7.0)	**< 0.001**	
	MUNO	52.6 (7.0)			53.4 (7.4)			53.1 (7.4)		
**Nutri-Score**	MHO	10.8 (2.8)	0.549	0.063	10.5 (2.9)	0.398	0.236	10.9 (2.7)	0.243	0.454
	MUO	10.6 (2.8)			10.7 (2.8)			10.6 (2.9)		
	MHNO	10.7 (2.9)	**0.017**		10.5 (2.9)	0.088		10.6 (2.9)	0.756	
	MUNO	10.3 (2.9)			10.8 (2.9)			10.5 (2.9)		

Abbreviations: DASH: Dietary Approaches to Stop Hypertension, DII: Dietary Inflammatory Index, HEI: Healthy Eating Index, E-DII: Energy-Adjusted Dietary Inflammatory Index, MHNO: Metabolically Healthy Non-Obese; MHO: Metabolically Healthy Obese, MUNO: Metabolically Unhealthy Non-Obese, MUO: Metabolically Unhealthy Obese, PDI: Plant-Based Diet Index, hPDI: Healthful Plant-Based Diet Index, uPDI: Unhealthful Plant-Based Diet Index

Values presented as means ± SD. Statistical analyses were conducted using an independent samples t-test

*Inter group comparison using one-way analysis of variance

Significant p-values are highlighted in **bold**

### Logistic regression analysis: MHO

**Fig. 1:**
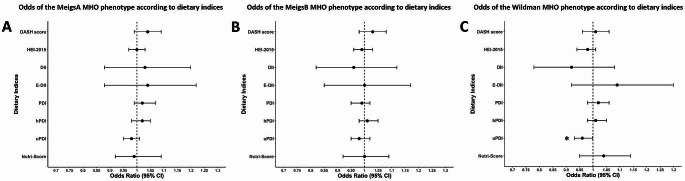
Odds of the MHO phenotype according to dietary indices. (Abbreviations: DASH: Dietary Approaches to Stop Hypertension, DII: Dietary Inflammatory Index, HEI: Healthy Eating Index, E-DII: Energy-Adjusted Dietary Inflammatory Index, MHO: Metabolically Healthy Obese, PDI: Plant-Based Diet Index, hPDI: Healthful Plant-Based Diet Index, uPDI: Unhealthful Plant-Based Diet. **A**. Odds of the MeigsA MHO phenotype according to dietary indices from fully adjusted (Model 3) logistic regression analysis. **B**. Odds of the MeigsB MHO phenotype according to dietary indices from fully adjusted (Model 3) logistic regression analysis. **C**. Odds of the Wildman MHO phenotype according to dietary indices from fully adjusted (Model 3) logistic regression analysis. All models adjusted for age, sex, energy intake, smoking status, alcohol use and physical activity. The E-DII and Nutri-Score were not adjusted for energy intake, as these scores already take energy into account. *p < 0.05.)

The results of logistic regression analyses, which examined dietary pattern associations with the MHO phenotype, are graphically presented in Fig. 1 for fully adjusted models, with numerical results for all models provided in Supplementary Table S2. In crude models, a one-unit increase in healthier diet scores, characterised by higher DASH, PDI and hPDI scores, was favourably associated with the MHO phenotype: DASH score (MeigsB: OR = 1.04, 95% CI: 1.01–1.08, *p* = 0.012), PDI (MeigsA: OR = 1.03, 95% CI: 1.00–1.06, *p* = 0.046), hPDI (MeigsB: OR = 1.03, 95% CI: 1.01–1.06, *p* = 0.018), whereas a one-unit increase in pro-inflammatory and unhealthful PBD scores, characterised by higher DII and uPDI scores, respectively, was inversely associated with the MHO phenotype: DII (MeigsA: OR = 0.90, 95% CI: 0.81–1.00, *p* = 0.044, Wildman: OR = 0.86, 95% CI: 0.76–0.96, *p* = 0.01) and uPDI (MeigsA: OR = 0.97, 95% CI: 0.95–0.99, *p* = 0.014, Wildman: OR = 0.97, 95% CI: 0.97–0.99, *p* = 0.016). After adjustment for age and sex, inverse associations for the DII (Wildman: OR = 0.87, 95% CI: 0.77–0.98, *p* = 0.021) and uPDI (Wildman: OR = 0.97, 95% CI: 0.95–1.00, *p* = 0.033) with MHO persisted. In fully adjusted models, only the association between the uPDI and decreased odds of MHO remained significant (Wildman: OR = 0.96, 95% CI: 0.93–1.00, *p* = 0.038), but did not persist after correction for multiple testing.

### Logistic regression analysis: MHNO

**Fig. 2:**
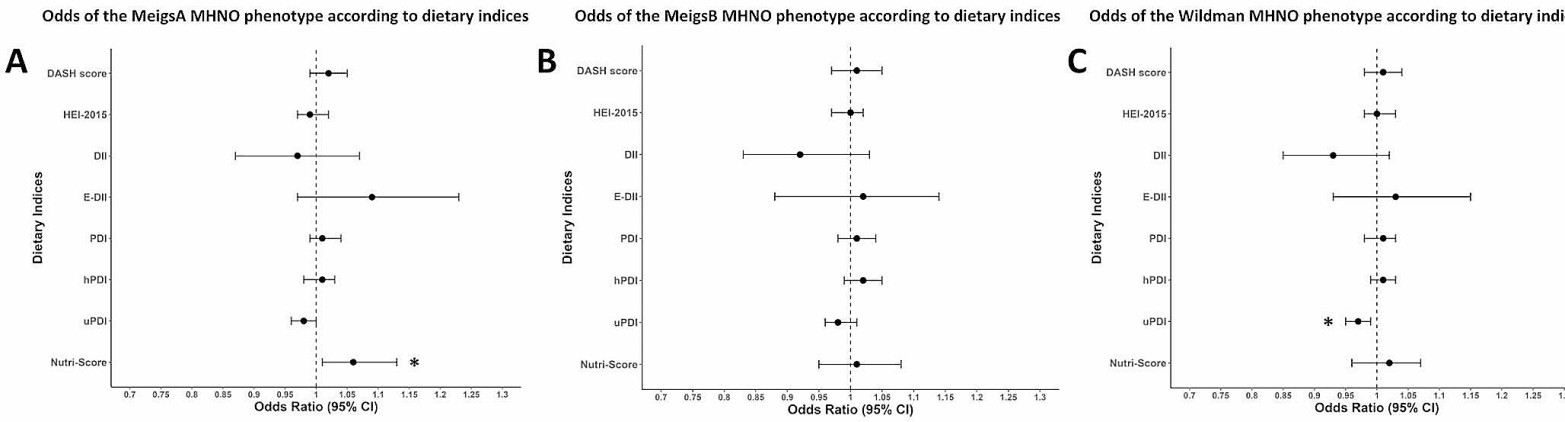
Odds of the MHNO phenotype according to dietary indices. (Abbreviations: DASH: Dietary Approaches to Stop Hypertension, DII: Dietary Inflammatory Index, HEI: Healthy Eating Index, E-DII: Energy-Adjusted Dietary Inflammatory Index, MHNO: Metabolically Healthy Non-Obese, PDI: Plant-Based Diet Index, hPDI: Healthful Plant-Based Diet Index, uPDI: Unhealthful Plant-Based Diet. **A**. Odds of the MeigsA MHNO phenotype according to dietary indices from fully adjusted (Model 3) logistic regression analysis. **B**. Odds of the MeigsB MHNO phenotype according to dietary indices from fully adjusted (Model 3) logistic regression analysis. **C**. Odds of the Wildman MHNO phenotype according to dietary indices from fully adjusted (Model 3) logistic regression analysis. All models adjusted for age, sex, energy intake, smoking status, alcohol use and physical activity. The E-DII and Nutri-Score were not adjusted for energy intake, as these scores already take energy into account. *p < 0.05

The results of logistic regression analyses, which examined dietary pattern associations with the MHNO phenotype, are graphically presented in Fig. 2 for fully adjusted models, with numerical results for all models provided in Supplementary Table S3. In crude models, a one-unit increase in healthier diets, characterised by higher DASH and hPDI scores, was positively associated with the MHNO phenotype: DASH score (MeigsB: OR = 1.04, 95% CI: 1.01–1.06, *p* = 0.006, Wildman: OR = 1.03, 95% CI: 1.00–1.05, *p* = 0.017) and hPDI (MeigsB: OR = 1.03, 95% CI: 1.01–1.05, *p* = 0.003). In contrast, a one-unit increase in pro-inflammatory and unhealthful PBD scores, characterised by higher DII and uPDI scores, respectively, was inversely associated with the MHNO phenotype: DII (MeigsB: OR = 0.90, 95% CI: 0.83–0.98, *p* = 0.013, Wildman: OR = 0.90, 95% CI: 0.84–0.96, *p* = 0.002) and uPDI (MeigsA: OR = 0.98, 95% CI: 0.97–1.00, *p* = 0.035, MeigsB: OR = 0.97, 95% CI: 0.95–0.99, *p* < 0.001 and Wildman: OR = 0.96, 95% CI: 0.95–0.98, *p* < 0.001). A one-unit increase in Nutri-Score, indicative of a less nutritious diet, was also associated with the MHNO phenotype (MeigsA: OR = 1.05, 95% CI: 1.01–1.09, *p* = 0.018). After adjustment for age and sex, healthier diet score associations with the MHNO phenotype persisted for the hPDI (MeigsB: OR = 1.03, 95% CI: 1.01–1.05, *p* = 0.012), along with the association between unhealthful PBD scores and decreased odds of MHNO: uPDI (MeigsB: OR = 0.98, 95% CI: 0.95–0.99, *p* = 0.023, Wildman: OR = 0.97, 95% CI: 0.96–0.99, *p* < 0.001). The association between the MHNO phenotype and Nutri-Score persisted in an age- and sex-adjusted model (MeigsA: OR = 1.06, 95% CI: 1.01–1.10, *p* = 0.011), whereas DASH score (MeigsB and Wildman), DII (MeigsB and Wildman) and uPDI (MeigsA) associations were attenuated. In fully adjusted models, only the uPDI (Wildman: OR = 0.97, 95% CI: 0.95–0.99, *p* = 0.006) and Nutri-Score (MeigsA: OR = 1.06, 95% CI: 1.01–1.13, *p* = 0.033) associations remained significant, but did not withstand correction for multiple testing.

### Sensitivity analyses

Table 3 shows results from fully adjusted logistic regression analyses which examined dietary pattern associations with MHO and MHNO phenotypes, but which excluded participants with implausible energy intakes. Regarding the MHO phenotype, the association with the uPDI (Wildman) was in the same direction as the main results (OR = 0.97, 95% CI: 0.93–1.00, *p* = 0.073). The uPDI and Nutri-Score associations with the MHNO phenotype persisted: uPDI (Wildman: OR = 0.97, 95% CI: 0.95–0.99, *p* = 0.004) and Nutri-Score (MeigsA: OR = 1.07, 95% CI: 1.01–1.14, *p* = 0.017).

**Table 3:** Odds of the MHO phenotype and MHNO phenotype according to dietary indices – excluding participants with implausible energy intake based on sex-specific cut-offs

MHO															
	DASH score	HEI-2015		DII		E-DII*		PDI		hPDI		uPDI		Nutri-Score*	
OR (95% CI)	p-value	OR (95% CI)	p-value	OR (95% CI)	p-value	OR (95% CI)	p-value	OR (95% CI)	p-value	OR (95% CI)	p-value	OR (95% CI)	p-value	OR (95% CI)	p-value
**MeigsA**															
1.03 (0.98–1.08)	0.233	1.00 (0.97–1.03)	0.966	1.02 (0.87–1.19)	0.817	1.03 (0.87–1.20)	0.754	1.02 (0.98–1.06)	0.292	1.01 (0.97–1.04)	0.637	0.99 (0.95–1.02)	0.388	0.99 (0.90–1.08)	0.737
**MeigsB**															
1.03 (0.98–1.08)	0.243	1.00 (0.96–1.03)	0.749	0.95 (0.81–1.11)	0.529	0.98 (0.83–1.17)	0.855	0.98 (0.95–1.02)	0.416	1.01 (0.98–1.05)	0.546	0.98 (0.95–1.01)	0.217	0.99 (0.91–1.08)	0.802
**Wildman**															
1.00 (0.95–1.05)	0.957	0.98 (0.95–1.02)	0.263	0.92 (0.78–1.09)	0.337	1.12 (0.93–1.35)	0.225	1.01 (0.97–1.06)	0.594	1.00 (0.96–1.04)	0.941	0.97 (0.93–1.00)	0.073	1.04 (0.95–1.14)	0.422
**MHNO**															
	**DASH score**	**HEI-2015**		**DII**		E-DII*		**PDI**		**hPDI**		**uPDI**		Nutri-Score*	
**OR (95% CI)**	**p-value**	**OR (95% CI)**	**p-value**	**OR (95% CI)**	**p-value**	**OR (95% CI)**	**p-value**	**OR (95% CI)**	**p-value**	**OR (95% CI)**	**p-value**	**OR (95% CI)**	**p-value**	**OR (95% CI)**	**p-value**
**MeigsA**															
1.01 (0.98–1.05)	0.417	1.00 (0.97–1.02)	0.655	0.97 (0.87–1.07)	0.494	1.13 (1.00–1.27)	0.057	1.02 (0.99–1.04)	0.281	1.00 (0.98–1.03)	0.746	0.98 (0.96–1.01)	0.123	1.07 (1.01–1.14)	**0.017**
**MeigsB**															
1.01 (0.97–1.05)	0.571	1.00 (0.97–1.03)	0.952	0.91 (0.81–1.02)	0.09	0.99 (0.87–1.13)	0.906	1.01 (0.98–1.05)	0.68	1.02 (0.99–1.04)	0.172	1.02 (1.00–1.05)	0.117	1.03 (0.96–1.10)	0.39
**Wildman**															
1.01 (0.98–1.05)	0.433	1.01 (0.99–1.03)	0.55	0.92 (0.84–1.02)	0.102	1.07 (0.95–1.20)	0.255	1.01 (0.99–1.04)	0.447	1.01 (0.98–1.04)	0.417	0.97 (0.95–0.99)	**0.004**	1.03 (0.97–1.09)	0.314

Logistic regression of metabolic obese phenotypes (dependent variable) and dietary score (independent variable) adjusted for age, sex, energy intake, smoking status, alcohol use and physical activity

Abbreviations: DASH: Dietary Approaches to Stop Hypertension, DII: Dietary Inflammatory Index, HEI: Healthy Eating Index, E-DII: Energy-Adjusted Dietary Inflammatory Index, MHNO: Metabolically Healthy Non-Obese; MHO: Metabolically Healthy Obese, MUNO: Metabolically Unhealthy Non-Obese, MUO: Metabolically Unhealthy Obese, PDI: Plant-Based Diet Index, hPDI: Healthful Plant-Based Diet Index, uPDI: Unhealthful Plant-Based Diet Index

Implausible energy intakes were defined as < 500 kcal/d and > 3,500 kcal/d for females, and < 800 kcal/d and > 4,000 kcal/d for males

*Excluding adjustment for energy intake, as energy intake is already accounted for in the calculation of these indices

Significant p-values are highlighted in **bold**

## Discussion

Using a random sample of 2,040 middle- to older-aged adults, this study evaluated relationships between dietary patterns, characterising dietary quality (DASH score, HEI-2015 and Nutri-Score), dietary inflammation (DII and E-DII) and PBD quality (PDI, hPDI and uPDI), and MH phenotypes, using MH definitions by MeigsA, MeigsB and Wildman. In age- and sex-adjusted models, a more unhealthful PBD and more pro-inflammatory diet, characterised by higher uPDI and DII scores, respectively, was associated with a decreased likelihood of MHO (Wildman). Conversely, greater adherence to a more PBD and a higher quality diet, characterised by higher PDI (MeigsA), hPDI and DASH scores (MeigsB), respectively, was associated with an increased odds of MHO in crude models. Among adults without obesity, higher uPDI and DII scores (MeigsB and Wildman) were also associated with a decreased likelihood of MH in an age- and sex- adjusted model and crude model, respectively. Conversely, higher hPDI scores (MeigsB) and Nutri-Score values (MeigsA) were associated with an increased odds of MH in age- and sex-adjusted models. In crude models, higher DASH scores (MeigsB, Wildman) were also associated with an increased likelihood of MH. The negative uPDI associations with both MHO and MHNO (Wildman) and positive Nutri-Score associations with MHNO (MeigsA) persisted in fully adjusted models and were generally robust to sensitivity analyses. While the finding for higher Nutri-Score values and increased likelihood of MH was unexpected, collectively, these results suggest that certain dietary patterns are associated with MH phenotypes, with healthier dietary patterns being positively associated with MH and unhealthier dietary patterns being negatively associated with MH.

Relationships between PDIs and MH status have been observed in previous research. A study of 289 Iranian women found that individuals with higher hPDI scores had a lower risk of MUO [32], while a recent study of 203 adolescents found unhealthful PBDs to be associated with an increased likelihood of MUO [33]. Higher uPDI scores have also been associated with abdominal obesity, higher fasting glucose levels and dyslipidaemia [34], along with lipoprotein-related insulin resistance [35]. One possible explanation for the unfavourable metabolic effects of unhealthful PBDs is the increased consumption of high-sugar plant-based foods, which can increase the risk of weight gain and negatively impact blood glucose control and lipid metabolism [36]. In support of this, a recent study of 347 adults reported a direct association between unhealthful PBDs and an increased odds of hyperglycaemia, possibly due to the high glycaemic index of these foods, which have a propensity to increase visceral adiposity and impact on the development of metabolic syndrome [37]. These findings provide some mechanistic plausibility to the relationship between greater uPDI adherence and lower likelihood of MH status observed in our study, especially considering that metabolically healthy participants had a lower BMI and waist circumference than their metabolically unhealthy counterparts (Table 1). Furthermore, a diet higher in unhealthful plant foods will necessarily displace healthful plant foods (e.g., whole grains) that positively affect biomarkers of MH [38]. Considering this, it is unsurprising that unhealthful PBDs have been found to be associated with a greater risk of cardiometabolic diseases in previous large cohort studies [24, 39, 40].

Healthful PBDs have been shown to be associated with more favourable inflammatory, lipid and lipoprotein profiles [35, 41], whereas unhealthful PBDs are associated with the reverse [35, 41, 42]. A reason for this may be that diets consisting of fruits, vegetables and legumes are also high in a range of nutrients and antioxidants, which may help prevent the low-grade systemic inflammation associated with increased adiposity [43]. Healthful PBDs are also typically abundant in polyphenols, low in saturated fatty acids and high in polyunsaturated fatty acids, which can reduce the accessibility of free fatty acids for adipose tissue uptake, help improve insulin sensitivity, and reduce the quantity of circulating LDL particles via upregulation of LDL receptor expression [44, 45]. In addition, healthful PBDs are also high in fibre, which can reduce concentrations of inflammatory biomarkers such as c-reactive protein (CRP), as well as pro-atherogenic cholesterol concentrations [23]. These nutritional characteristics provide context to previous research demonstrating higher adherence to the hPDI to be associated with reduced cardiometabolic disease risk [24, 39, 40, 46].

We also observed significant associations between higher DII scores, reflective of a more pro-inflammatory diet and a decreased likelihood of MHO/MHNO in unadjusted models (MeigsB and Wildman for both MHO/MHNO), which persisted in an age- and sex-adjusted model for those living with obesity (Wildman). A narrative review on the DII and risk of non-communicable diseases concluded that a more pro-inflammatory diet was associated with an increased risk of CVD and cancer [47], and these findings were echoed in another study which found that greater adherence to a pro-inflammatory dietary pattern was associated with a higher likelihood of MUO and cardiometabolic abnormalities [19]. A previous study in the Mitchelstown cohort reported that higher DII and E-DII scores, indicative of a more pro-inflammatory diet, were associated with greater concentrations of CRP, complement component 3 (C3), interleukin 6 (IL-6), tumour necrosis factor alpha (TNF-α), white blood cell counts, monocytes (DII only), neutrophils, the neutrophil-to-lymphocyte ratio and resistin [18]. In addition, higher E-DII scores were associated with pro-atherogenic lipoprotein profiles [48] and MHO and MHNO individuals had more favourable inflammatory profiles than their MUO and MUNO counterparts [49]. Collectively, these findings suggest a protective effect of anti-inflammatory diets on MH status.

Interestingly, we found higher Nutri-Score values, reflecting a diet of lower nutritional quality, to be associated with an increased likelihood of MHNO (MeigsA) in a fully adjusted model. To our knowledge, no studies have investigated Nutri-Score values in relation to MH phenotypes, making comparison difficult. This finding was unique to the MHNO phenotype and only observed in one definition, so cannot be taken definitively. In another study of the same cohort, poorer dietary quality, defined using Nutri-Score rating, was found to be positively associated with inflammatory biomarkers, including CRP, C3, IL-6 and TNF-α [25]. Another large study of over 500,000 participants reported greater all-cause mortality risk for those with higher Nutri-Score values [50], highlighting the requirement for further research into this particular dietary score.

### Strengths and limitations

The main strengths of this study are the relatively large sample size and equal representation of both sexes. The inclusion of several dietary indices permits comparison of different aspects of habitual diet. Also, as there is no gold standard definition for MH, we examined three commonly used definitions. Several potential confounders were adjusted for and correction for multiple testing and sensitivity analyses were performed. The main limitation is the inability to make an inference about causal and temporal relationships due to the cross-sectional nature of the data. Furthermore, though we applied a stringent multiple hypothesis correction, it should be noted that although correcting for multiple testing reduces the probability of false positive findings, it may also increase the probability of false negative results. In addition, self-reported dietary questionnaires were used which are subject to recall and reporting bias. Also, while a number of potential confounders were adjusted for, residual confounding from inaccurate measurement of variables cannot be ruled out. Another limitation of our study is the lack of diversity in the diet of the sampled population as homogeneity of diet will decrease the likelihood of detecting a true association between diet and MH phenotypes. Finally, our data were collected from one geographic source, which may not be reflective of the general population and therefore may not be generalisable.

## Conclusion

Collectively, these findings suggest that specific dietary patterns are associated with MH phenotypes, with healthier dietary patterns positively influencing MH and unhealthier dietary patterns negatively influencing MH. In particular, adherence to a more unhealthful plant-based diet was associated with a decreased likelihood of being metabolically healthy (defined by Wildman), irrespective of BMI classification. These findings therefore have significance for public health nutrition and highlight the importance of shifting towards healthful (as opposed to unhealthful) PBDs to safeguard the metabolic health of individuals, as well as the health of the planet [11]. We must acknowledge that the concept of MHO is an appealing but controversial one; it is not a benign condition, with individuals still at an increased risk of developing T2DM and CVD compared to their MHNO counterparts (albeit a reduced risk compared to their MUO counterparts) [51]. Therefore, there is a clear requirement to fully understand the metabolic risk that those living with obesity are exposed to, beyond the simple BMI classification, as well as to standardise the quantification of MHO. Future research should investigate associations between similar dietary indices and MHO in populations with more diverse diets to better understand the relationship between dietary patterns and MHO.

## Electronic supplementary material

Below is the link to the electronic supplementary material.

Supplementary Material 1

Supplementary Material 2

## Data Availability

Data described in the manuscript are freely available to other researchers upon request.
