# Supplementary material for: Plant-based diet adherence is associated with metabolic health status in adults living with and without obesity
Source: Eur J Nutr. 2024 May 16;63(6):2235–46. doi: 10.1007/s00394-024-03399-7 (PMC11377579; doi:10.1007/s00394-024-03399-7)
Supplement: Supplementary file 2 — Supplementary Material 2 [file 394_2024_3399_MOESM2_ESM.docx]

**Supplementary File**

Title: Plant-based diet adherence is associated with metabolic health status in adults living with and without obesity

Mags T. Carey, University College Dublin, School of Public Health, Physiotherapy and Sports Science, Dublin 4, Ireland

Seán R. Millar, HRB Centre for Health and Diet Research, School of Public Health, University College Cork, Cork, Ireland.

Patrick S. Elliott, Institute of Food and Health, School of Agriculture and Food Science, University College Dublin, Dublin 4, Ireland.

Pilar Navarro, School of Public Health, Physiotherapy and Sports Science, University College Dublin, Dublin 4, Ireland.

Janas M. Harrington, HRB Centre for Health and Diet Research, School of Public Health, University College Cork, Cork, Ireland.

Ivan J. Perry, HRB Centre for Health and Diet Research, School of Public Health, University College Cork, Cork, Ireland.

Catherine M. Phillips, School of Public Health, Physiotherapy and Sports Science, University College Dublin, Dublin 4, Ireland.

**Dietary Definitions**

**Dietary Approaches to Stop Hypertension (DASH) Score**

The DASH diet is rich in fruits, vegetables, wholegrains and low-fat dairy to prevent and control hypertension. To create the DASH score, the consumption of eight food components (fruits, vegetables, nuts and legumes, low-fat dairy products, wholegrains, sodium, sweetened beverages and red and processed meats) was divided into quintiles and participants were classified according to their intake ranking [1]. Consumption of healthy food components was rated on a scale of one to five; the higher the score, the more frequent the consumption of that food. Less healthy dietary constituents, where low consumption is desired, were scored on a reverse scale, with lower consumption receiving higher scores. Component scores were summed, and an overall DASH score was calculated for each person. Scores ranged from 13 to 45, according to adherence to the diet [2], with lower scores representing poorer, and higher scores indicating better diet quality.

**Healthy Eating Index-2015 (HEI-2015)**

The HEI is a measure for assessing dietary quality, specifically with regard to the degree with which a set of foods align with the Dietary Guidelines for Americans [3]. The HEI-2015 is comprised of 13 separate components, reflecting food groups and dietary recommendations, with scores ranging from 0 to 100 in order of compliance with recommendations [4]. HEI-2015 scores ranged from 21 to 62 for this study.

### **Dietary Inflammatory Index (DII) and Energy Adjusted Dietary Inflammatory Index (E-DII)**

The DII is an established method used to evaluate the influence of diet on inflammatory biomarkers (namely IL-1β, IL-4, IL-6, IL-10, TNF-α, and CRP), based on 45 different food components, with pro-inflammatory diets having higher scores, and anti-inflammatory diets scoring lower [5], which has been previously described for this cohort [1].

A total of 26 of the 45 possible food parameters were used for the DII calculation based on the food frequency questionnaire (FFQ) in this study and these were as follows: carbohydrate, protein, fat, alcohol, fibre, cholesterol, saturated fat, monounsaturated fat, polyunsaturated fat, niacin, thiamin, riboflavin, vitamin B12, vitamin B6, iron, magnesium, zinc, selenium, vitamin A, vitamin C, vitamin D, vitamin E, folic acid, onion, garlic and tea. The E-DII minimises the effect of energy intake by dividing DII by total energy intake. DII scores may range from -8.87 (strongly anti-inflammatory) to 7.98 (highly pro-inflammatory). The data for this research had DII scores ranging from -3.88 to 3.70 and E-DII scores ranging from -4.15 to 3.84.

**Plant-Based Dietary Indices (PDIs, hPDIs and uPDIs)**

Plant based dietary indices comprise of an overall plant-based dietary index (PDI), a healthful plant-based diet index (hPDI) representing greater consumption of high-quality plant foods and an unhealthful plant-based diet index (uPDI) representing greater consumption of less healthy plant foods, based on 18 food groups [6]. Plant foods were classified as ‘healthy’ or ‘unhealthy’ based on existing knowledge of the associations between these foods and chronic disease [7]. Observed ranges in the current study were 31 to 72 for the PDI, 31 to 76 for the hPDI and 30 to 75 for the uPDI. For the PDI, higher scores represent a more plant-based diet (PBD). For the hPDI, higher scores represent a more healthful PBD. For the uPDI, higher scores represent a more unhealthful PBD.

**Nutri-Score**

The Food Standards Agency Nutrient Profiling System forms the basis of the five-colour front-of-pack labelling system known as Nutri-Score, which categorises foods and drinks as ‘healthier’ and ‘less healthy’. The Nutri-Score, which punctuates the amount of nutrients per 100 grams of product, was calculated for all foods and beverages in the Mitchelstown FFQ [8]. Nutri-Score values are on a discrete continuous scale and may range from -15 (most healthy) to +40 (least healthy). Nutri-Score values for this study ranged from -2.20 to 17.92.

**Table S1: Criteria used to define metabolic health status**

|  | | | |
| --- | --- | --- | --- |
| **MH Definition** | **MeigsA** | **MeigsB** | **Wildman** |
| **BP** (mmHg) | SBP >=130 and DBP >=85 or treatment |  | SBP >=130 and DBP >=85 or treatment |
| **TG** (mmol/L) | >=1.70 |  | >=1.70 |
| **HDL-C** (mmol/L) | Males <1.04 Females <1.30 |  | Males <1.04  Females <1.30  or treatment |
| **FPG** (mmol/L) | >=5.60 or treatment |  | >=5.55 or treatment |
| **HOMA-IR** |  | <75^th^ percentile | >90^th^ percentile |
| **Other** | Males >102 cm  Females >88cm |  | CRP >90^th^ percentile |
| **MH Criteria** | <3 of above | All of above | <2 of above |

Abbreviations: BP: Blood pressure, CRP: c-reactive protein, DBP: diastolic blood pressure, FPG: fasting plasma glucose, HDL-C: high density lipoprotein cholesterol, HOMA-IR: Homeostatic Model Assessment for Insulin Resistance, LDL: low density lipoprotein, MH: metabolic health, SBP: systolic blood pressure; TG: triglycerides

**Table S2: Odds of the MHO phenotype according to dietary indices**

|  | **DASH score** | | **HEI-2015** | | **DII** | | **E-DII*** | | **PDI** | | | **hPDI** | | **uPDI** |  | **Nutri-Score*** | |
| --- | --- | --- | --- | --- | --- | --- | --- | --- | --- | --- | --- | --- | --- | --- | --- | --- | --- |
|  | **OR (95% CI)** | **p-value** | **OR (95% CI)** | **p-value** | **OR (95% CI)** | **p-value** | **OR (95% CI)** | **p-value** | **OR (95% CI)** | | **p-value** | **OR (95% CI)** | **p-value** | **OR (95% CI)** | **p-value** | **OR (95% CI)** | **p-value** |
|  |  |  |  |  |  |  |  |  | |  |  |  |  |  |  |  |  |
|  |  |  |  |  |  |  |  | **MeigsA** | |  |  |  |  |  |  |  |  |
|  |  |  |  |  |  |  |  |  |  | |  |  |  |  |  |  |  |
| **Model 1** | 1.03  (0.99–1.06) | 0.143 | 1.01  (0.99–1.04) | 0.358 | 0.90  (0.81–1.00) | 0.053 | 1.01  (0.90–1.14) | 0.876 | 1.03  (1.00–1.06) | | **0.046** | 1.00  (0.97–1.02) | 0.766 | 0.98 (0.96–1.01) | 0.168 | 1.02  (0.96–1.08) | 0.549 |
| **Model 2** | 1.03  (0.99–1.06) | 0.165 | 1.01  (0.98–1.03) | 0.487 | 0.92  (0.82–1.02) | 0.124 | 1.01  (0.90–1.14) | 0.849 | 1.03  (1.00–1.06) | | 0.084 | 1.00 (0.98–1.03) | 0.933 | 0.99 (0.96–1.01) | 0.246 | 1.02 (0.96–1.08) | 0.533 |
| **Model 3** | 1.04 (0.99–1.09) | 0.158 | 1.00 (0.97–1.03) | 0.906 | 1.03 (0.88–1.20) | 0.718 | 1.04 (0.88–1.22) | 0.65 | 1.02 (0.99–1.07) | | 0.228 | 1.02 (0.98–1.05) | 0.373 | 0.98 (0.95–1.01) | 0.212 | 0.99 (0.92–1.09) | 0.996 |
| **MeigsB** | | | | | | | | | | | | | | | | | |
|  |  |  |  |  |  |  |  |  |  | |  |  |  |  |  |  |  |
| **Model 1** | 1.04 (1.01–1.08) | **0.012** | 1.00 (0.98–1.03) | 0.77 | 0.90 (0.81–1.00) | **0.044** | 0.91 (0.81–1.02) | 0.097 | 1.01 (0.98–1.04) | | 0.591 | 1.03 (1.01–1.06) | **0.018** | 0.97 (0.95–0.99) | **0.014** | 0.98 (0.92–1.03) | 0.397 |
| **Model 2** | 1.03 (0.99–1.06) | 0.163 | 1.00  (0.97–1.02) | 0.901 | 0.92 (0.82–1.02) | 0.11 | 0.95 (0.84–1.08) | 0.433 | 1.00 (0.98–1.03) | | 0.819 | 1.02 (1.00–1.05) | 0.065 | 0.98 (0.95–1.00) | 0.052 | 0.99 (0.93–1.05) | 0.716 |
| **Model 3** | 1.03 (0.98–1.08) | 0.205 | 0.99 (0.96–1.03) | 0.697 | 0.96 (0.82–1.12) | 0.591 | 1.00 (0.85–1.17) | 0.985 | 0.99 (0.95–1.02) | | 0.439 | 1.01 (0.98–1.05) | 0.457 | 0.98 (0.95–1.02) | 0.316 | 1.00 (0.92–1.09) | 0.961 |
| **Wildman** | | | | | | | | | | | | | | | | | |
|  |  |  |  |  |  |  |  |  |  | |  |  |  |  |  |  |  |
| **Model 1** | 1.02 (0.99–1.06) | 0.226 | 1.00 (0.98–1.03) | 0.882 | 0.86 (0.76–0.96) | **0.01** | 1.00 (0.88–1.14) | 0.975 | 1.03 (1.00–1.06) | | 0.068 | 1.00 (0.98–1.03) | 0.804 | 0.97 (0.97–0.99) | **0.016** | 1.04 (0.97–1.11) | 0.242 |
| **Model 2** | 1.01 (0.97–1.05) | 0.528 | 1.00  (0.97–1.03) | 0.926 | 0.87 (0.77–0.98) | **0.021** | 1.03 (0.91–1.18) | 0.629 | 1.03 (1.00–1.06) | | 0.099 | 1.00 (0.97–1.03) | 0.992 | 0.97 (0.95–1.00) | **0.033** | 1.05 (0.98–1.12) | 0.15 |
| **Model 3** | 1.01 (0.96–1.06) | 0.738 | 0.98 (0.94–1.01) | 0.222 | 0.92 (0.78–1.08) | 0.293 | 1.09 (0.92–1.30) | 0.338 | 1.02 (0.98–1.06) | | 0.418 | 1.01 (0.98–1.05) | 0.579 | 0.96 (0.93–1.00) | **0.038** | 1.04 (0.95–1.14) | 0.385 |
|  | | | | | | | | | | | | | | | | | |

Abbreviations: DASH: Dietary Approaches to Stop Hypertension, DII: Dietary Inflammatory Index, HEI: Healthy Eating Index, E-DII: Energy-Adjusted Dietary Inflammatory Index, MHNO: Metabolically Healthy Non-Obese; MHO: Metabolically Healthy Obese, MUNO: Metabolically Unhealthy Non-Obese, MUO: Metabolically Unhealthy Obese, PDI: Plant-Based Dietary Index, hPDI: Healthful Plant-Based Diet Index, uPDI: Unhealthful Plant-Based Diet Index

Odds of metabolic obese phenotypes (dependent variable) according to dietary scores (independent variable) were calculated using logistic regression analysis.

Model 1: Unadjusted
Model 2: Adjusted for age and sex

Model 3: Fully adjusted for age, sex, energy intake, smoking status, alcohol use and physical activity

*Excluding adjustment for energy intake, as energy intake is already accounted for in the calculation of these indices.

Significant p-values are highlighted in **bold**.

**Table S3: Odds of the MHNO phenotype according to dietary indices**

|  | **DASH score** | | **HEI-2015** | | **DII** | | **E-DII*** | | **PDI** | | | **hPDI** | | **uPDI** |  | **Nutri-Score*** | |
| --- | --- | --- | --- | --- | --- | --- | --- | --- | --- | --- | --- | --- | --- | --- | --- | --- | --- |
|  | **OR (95% CI)** | **p-value** | **OR (95% CI)** | **p-value** | **OR (95% CI)** | **p-value** | **OR (95% CI)** | **p-value** | **OR (95% CI)** | | **p-value** | **OR (95% CI)** | **p-value** | **OR (95% CI)** | **p-value** | **OR (95% CI)** | **p-value** |
|  |  |  |  |  |  |  |  |  | |  |  |  |  |  |  |  |  |
|  |  |  |  |  |  |  |  | **MeigsA** | |  |  |  |  |  |  |  |  |
|  |  |  |  |  |  |  |  |  |  | |  |  |  |  |  |  |  |
| **Model 1** | 1.01 (0.99–1.03) | 0.41 | 1.00 (0.98–1.01) | 0.582 | 0.94 (0.88–1.01) | 0.107 | 1.05 (0.97–1.14) | 0.204 | 1.01 (0.99–1.03) | | 0.16 | 1.00 (0.98–1.01) | 0.539 | 0.98 (0.97–1.00) | **0.035** | 1.05 (1.01–1.09) | **0.018** |
| **Model 2** | 1.01 (0.99–1.03) | 0.398 | 0.99 (0.98–1.01) | 0.335 | 0.98 (0.91–1.06) | 0.604 | 1.07 (0.98–1.16) | 0.133 | 1.01 (0.99–1.03) | | 0.485 | 1.00 (0.98–1.02) | 0.908 | 0.99 (0.97–1.01) | 0.157 | 1.06 (1.01–1.10) | **0.011** |
| **Model 3** | 1.02 (0.99–1.05) | 0.307 | 0.99 (0.97–1.02) | 0.575 | 0.97 (0.87–1.07) | 0.477 | 1.09 (0.97–1.23) | 0.141 | 1.01 (0.99–1.04) | | 0.404 | 1.01 (0.98–1.03) | 0.657 | 0.98 (0.96–1.00) | 0.076 | 1.06 (1.01–1.13) | **0.033** |
| **MeigsB** | | | | | | | | | | | | | | | | | |
|  |  |  |  |  |  |  |  |  |  | |  |  |  |  |  |  |  |
| **Model 1** | 1.04 (1.01–1.06) | **0.006** | 1.01 (0.99–1.03) | 0.185 | 0.90 (0.83–0.98) | **0.013** | 0.91 (0.83–1.00) | 0.052 | 1.02 (0.99–1.04) | | 0.168 | 1.03 (1.01–1.05) | **0.003** | 0.97 (0.95–0.99) | **<0.001** | 0.96 (0.92–1.01) | 0.088 |
| **Model 2** | 1.02 (0.99–1.05) | 0.205 | 1.01 (0.99–1.03) | 0.56 | 0.95 (0.87–1.03) | 0.193 | 0.97 (0.88–1.06) | 0.493 | 1.01 (0.99–1.03) | | 0.417 | 1.03 (1.01–1.05) | **0.012** | 0.98 (0.95–0.99) | **0.023** | 0.98 (0.94–1.03) | 0.433 |
| **Model 3** | 1.01 (0.97–1.05) | 0.66 | 1.00 (0.97–1.02) | 0.9 | 0.92 (0.83–1.03) | 0.16 | 1.02 (0.88–1.14) | 0.992 | 1.01 (0.98–1.04) | | 0.622 | 1.02 (0.99–1.05) | 0.16 | 0.98 (0.96–1.01) | 0.175 | 1.01 (0.95–1.08) | 0.764 |
| **Wildman** | | | | | | | | | | | | | | | | | |
|  |  |  |  |  |  |  |  |  |  | |  |  |  |  |  |  |  |
| **Model 1** | 1.03 (1.00–1.05) | **0.017** | 1.01 (0.99–1.04) | 0.485 | 0.90 (0.84–0.96) | **0.002** | 0.98 (0.91–1.06) | 0.639 | 1.02 (1.00–1.04) | | 0.06 | 1.01 (0.99–1.02) | 0.268 | 0.96 (0.95–0.98) | **<0.001** | 1.01 (0.97–1.05) | 0.756 |
| **Model 2** | 1.01 (0.99–1.04) | 0.268 | 1.00 (0.98–1.02) | 0.862 | 0.95 (0.88–1.02) | 0.13 | 1.03 (0.94–1.12) | 0.489 | 1.01 (0.99–1.03) | | 0.354 | 1.01 (0.99–1.03) | 0.202 | 0.97 (0.96–0.99) | **<0.001** | 1.02 (0.98–1.07) | 0.247 |
| **Model 3** | 1.01 (0.98–1.04) | 0.501 | 1.00 (0.98–1.03) | 0.807 | 0.93 (0.85–1.02) | 0.14 | 1.03 (0.93–1.15) | 0.562 | 1.01 (0.98–1.03) | | 0.565 | 1.01 (0.99–1.03) | 0.406 | 0.97 (0.95–0.99) | **0.006** | 1.02 (0.96–1.07) | 0.524 |
|  | | | | | | | | | | | | | | | | | |

Abbreviations: DASH: Dietary Approaches to Stop Hypertension, DII: Dietary Inflammatory Index, HEI: Healthy Eating Index, E-DII: Energy-Adjusted Dietary Inflammatory Index, MHNO: Metabolically Healthy Non-Obese; MHO: Metabolically Healthy Obese, MUNO: Metabolically Unhealthy Non-Obese, MUO: Metabolically Unhealthy Obese, PDI: Plant-Based Diet Index, hPDI: Healthful Plant-Based Diet Index, uPDI: Unhealthful Plant-Based Diet Index

Odds of metabolic obese phenotypes (dependent variable) according to dietary scores (independent variable) were calculated using logistic regression analysis.

Model 1: Unadjusted
Model 2: Adjusted for age and sex

Model 3: Fully adjusted for age, sex, energy intake, smoking status, alcohol use and physical activity

*Excluding adjustment for energy intake, as energy intake is already accounted for in the calculation of these indices.

Significant p-values are highlighted in **bold**.

**References**

1. Millar SR, Navarro P, Harrington JM, Shivappa N, Hebert JR, Perry IJ, Phillips C.M. (2022) Dietary score associations with markers of chronic low-grade inflammation: a cross-sectional comparative analysis of a middle- to older-aged population. https://link.springer.com/article/10.1007/s00394-022-02892-1. Accessed 05/05/2023

2. Fung TT, Chiuve SE, McCullough ML (2008) Adherence to a DASH-Style Diet and Risk of Coronary Heart Disease and Stroke in Women. https://jamanetwork.com/journals/jamainternalmedicine/fullarticle/414155. Accessed 17/04/2023

3. USDA (2022) Healthy Eating Index. https://[www.fns.usda.gov/healthy-eating-index-hei](http://www.fns.usda.gov/healthy-eating-index-hei). Accessed 23/03/2023

4. Millar SR, Navarro P, Harrington JM, Perry IJ, Phillips CM (2021) Dietary Quality Determined by the Healthy Eating Index-2015 and Biomarkers of Chronic Low-Grade Inflammation: A Cross-Sectional Analysis in Middle-to-Older Aged Adults. https://[www.mdpi.com/2072-6643/13/1/222](http://www.mdpi.com/2072-6643/13/1/222). Accessed 25/10/2023

5. Shivappa N, Steck SE, Hurley TG, Hussey JR, Hebert JR (2013) Designing and developing a literature-derived, population-based dietary inflammatory index. https://[www.cambridge.org/core/journals/public-health-nutrition/article/designing-and-developing-a-literaturederived-populationbased-dietary-inflammatory-index/30BE2C2295CE93DC6B54F9F9AD50CC68](http://www.cambridge.org/core/journals/public-health-nutrition/article/designing-and-developing-a-literaturederived-populationbased-dietary-inflammatory-index/30BE2C2295CE93DC6B54F9F9AD50CC68). Accessed 12/03/2023

6. Elliott PS, Harrington JM, Millar SR, Otvos JD, Perry IJ, Phillips CM (2023) Plant-based diet indices and lipoprotein particle subclass profiles: A cross-sectional analysis of middle- to older-aged adults. https://[www.sciencedirect.com/science/article/pii/S0021915023050979](http://www.sciencedirect.com/science/article/pii/S0021915023050979). Accessed 05/05/2023

7. Satija A, Bhupathiraju SN, Rimm EB, Spiegelman D, Chiuve SE, Borgi L, Willett WC, Manson JE, Sun Q, Hu FB (2016) Plant-Based Dietary Patterns and Incidence of Type 2 Diabetes in US Men and Women: Results from Three Prospective Cohort Studies. https://journals.plos.org/plosmedicine/article?id=10.1371/journal.pmed.1002039. Accessed 12/2/2023

8. Millar SR, Navarro P, Harrington JM, Perry IJ, Phillips CM (2022) Associations between the Nutrient Profiling System Underlying the Nutri-Score Nutrition Label and Biomarkers of Chronic Low-Grade Inflammation: A Cross-Sectional Analysis of a Middle- to Older-Aged Population. https://[www.ncbi.nlm.nih.gov/pmc/articles/PMC9370507/](http://www.ncbi.nlm.nih.gov/pmc/articles/PMC9370507/). Accessed 11/05/2023
